# Fast Video Encryption Using the H.264 Error Propagation Property for Smart Mobile Devices

**DOI:** 10.3390/s150407953

**Published:** 2015-04-02

**Authors:** Yongwha Chung, Sungju Lee, Taewoong Jeon, Daihee Park

**Affiliations:** Department of Computer Information Science, Korea University, Sejong KS002, Korea; E-Mails: ychungy@korea.ac.kr (Y.C.); dhpark@korea.ac.kr (D.P.)

**Keywords:** video sensor network, selective encryption, smartphone, error propagation

## Abstract

In transmitting video data securely over Video Sensor Networks (VSNs), since mobile handheld devices have limited resources in terms of processor clock speed and battery size, it is necessary to develop an efficient method to encrypt video data to meet the increasing demand for secure connections. Selective encryption methods can reduce the amount of computation needed while satisfying high-level security requirements. This is achieved by selecting an important part of the video data and encrypting it. In this paper, to ensure format compliance and security, we propose a special encryption method for H.264, which encrypts only the DC/ACs of I-macroblocks and the motion vectors of P-macroblocks. In particular, the proposed new selective encryption method exploits the error propagation property in an H.264 decoder and improves the collective performance by analyzing the tradeoff between the visual security level and the processing speed compared to typical selective encryption methods (*i.e.*, I-frame, P-frame encryption, and combined I-/P-frame encryption). Experimental results show that the proposed method can significantly reduce the encryption workload without any significant degradation of visual security.

## 1. Introduction

In transmitting video data over Video Sensor Networks (VSNs), the video data can be sent to and from portable devices such as smartphones [1,2,3,4,5]. Frequently, multimedia data delivery raises issues around content ownership and privacy; therefore, protecting content is important in multimedia applications [6,7,8,9,10,11,12,13,14,15,16,17,18,19,20,21,22,23,24,25,26,27,28,29,30,31,32,33,34,35,36]. Since the size of multimedia data is large, efficient encryption methods for protecting content, while also satisfying real-time requirements, are necessary [3,4,5].

Multimedia encryption methods [6,7,8,9,10] based on the chaos cryptography theory [11] can serve to protect video contents. These methods have exploited the concept of scrambling image pixels with a secret key. In general, the compression overhead is considerably higher than the encryption overhead in such methods. With the raw video data in an uncompressed format, the selective encryption methods generally increase the size of the compressed bit stream; that is, compression efficiency is compromised. This is not desirable, because the main goal of compression is to decrease the data size. Therefore, in this paper, we focus on the compressed format scenario only [12,13,14,15,16,17,18,19,20,21,22,23,24,25,26,27,28,29,30,31,32,33,34,35,36]. That is, any standard decoder should be able to decode the encrypted video data, but the decoded video without decryption had to be invisible. This is a very important requirement, as it allows for certain features of the compression algorithm to be preserved. Therefore, it is necessary to develop an efficient method to encrypt the compressed video data for ensuring format compliance. The proposed new selective encryption scheme for compressed video data satisfies the following requirements:

- Security: The encrypted video data can ensure both confidentiality and integrity, and the user cannot completely decrypt the encrypted video data without a single shared key.
- Format compliance: The encrypted video streams are compliant with the compression specification, and compatible with the standard decoder.
- Consideration of limited computational complexity: The encryption and decryption processes take into account the limited computational resources and times of mobile handheld devices.

To compress the video data, we exploit the characteristics of H.264/AVC [1,2,3,4,5], which is one of the lossy video compression methods for removing spatial and temporal redundancy, and typically consists of the intra- and inter-mode. In these modes, a current macroblock refers to the previous macroblock and pixels. Thus, if the referenced information (*i.e.*, the previous macroblock and pixels) is lossy, the H.264 cannot completely decode the compressed video data. The aim of the proposed method is to decrease the workload of encrypting the macroblocks of the I- and P-frames, excluding the header, by exploiting the error propagation property of the H.264 standard. In this paper, to simultaneously ensure format compliance and security, we encrypt only the DC/ACs of I-macroblocks and the motion vectors of P-macroblocks. Further, we improve the collective performance by analyzing the tradeoff between the visual security level and the processing speed, and we also exploit the AES-CCM for ensuring both multimedia data confidentiality and integrity [37]. To the best of our knowledge, this is the first report on exploiting the error propagation property of the H.264 standard and analyzing the tradeoff between the visual security level and the processing speed. Experimental results show that the proposed method can reduce the workload of both the full encryption method and the typical selective encryption methods (*i.e.*, I-frame, P-frame encryption, and combined I-/P-frame encryption) without any significant degradation of visual security.

The remainder of the paper is structured as follows: Section 2 describes the selective encryption methods for video data. Section 3 describes the selective encryption of video by using the error propagation property of H.264 with AES-CCM. Section 4 and Section 5 provide the experimental results and conclusion, respectively.

## 2. Related Work

Multimedia data delivery raises issues around content ownership, privacy, and integrity; thus, protecting multimedia data is important in multimedia applications [6,7,8,9,10,11,12,13,14,15,16,17,18,19,20,21,22,23,24,25,26,27,28,29,30,31,32,33,34,35,36]. Selective encryption methods can reduce the amount of computation needed while satisfying high-level security requirements. Selective encryption methods can be implemented by either encryption-before-compression or encryption-during-compression [23]. If encryption is performed before compression, the compression performance may be degraded. In general, the compression overhead is considerably higher than the encryption overhead in such methods. With the raw video data in an uncompressed format, the selective encryption methods generally increase the size of the compressed bit stream; that is, compression efficiency is compromised. Thus, if the entire visual information should be concealed, these approaches are not the method of choice. If only smaller areas need to be concealed, encryption-before-compression can be used. For privacy preservation, the straight-forward solution would be to cut out the privacy endangering areas and code them independently and encrypt them afterwards.

Another solution is to encrypt image areas and encode the modified image, e.g., a permutation of positions of the pixels in the privacy-endangering areas has been proposed in [6,7,8,9,10] based on the chaos cryptography theory [11], which can serve to protect video contents. These methods have exploited the concept of scrambling image pixels with a secret key. However, this is not desirable because the main goal of compression is to decrease the data size. Therefore, in this paper, we focus on the compressed format scenario only [12,13,14,15,16,17,18,19,20,21,22,23,24,25,26,27,28,29,30,31,32,33,34].

In encryption-during-compression [12,13,14,15,16,17,18,19,20,21,22,23,24,25,26,27,28,29,30,31,32,33,34], every image is processed in blocks, starting with 16 × 16 macroblocks, and the macroblocks can be grouped in slices. Also, most commonly a slice consists of the macroblocks of an entire image. In an I-frame, intra-coding is permitted and all previously decoded reference pictures will not be used in the further decoding process. Many video compression methods have used lossy techniques to remove spatial and temporal redundancy [12,13,14,15,16,17,18,19,20,21,22,23,24,25,26,27,28,29,30,31,32,33,34], and typically consist of the intra- and inter-mode. In these modes, a current macroblock refers to the previous macroblock and pixels. Thus, if the referenced information (*i.e.*, the previous macroblock and pixels) is lossy, the H.264 cannot completely decode the compressed video data. In this paper, to simultaneously ensure format compliance and security, we encrypt only the DC/ACs of I-macroblocks and the motion vectors of P-macroblocks for transmitting video data securely over Video Sensor Networks (VSNs).

In addition, to measure the security level, the visual security has been represented as PSNR or MSE in some researches for selective encryptions [28,29,30,31,32]. Also, the execution time or encrypted data size can be measured to verify the improvement of speed for selective encryptions. To the best of our knowledge, a study of collective performance by analyzing the tradeoff between the visual security level and the processing speed has not been reported. One of the most famous papers in analyzing a performance tradeoff is the collective analysis of area-time complexity for VLSI [38,39,40]. Thus, we apply the idea of collective analysis to represent the performance tradeoff between the visual security and the processing speed.

## 3. Proposed Selective Encryption for H.264

In this paper, we encrypt only the DC/ACs of I-macroblocks and the MVs of P-macroblocks in order to exploit the error propagation of the H.264 standard, with AES-CCM [37] for ensuring both the confidentiality and the integrity of video data. Further, we improve the collective performance by analyzing the tradeoff between the visual security level and the processing speed.

### 3.1. Error Propagation Property of H.264

The digital video data can be compressed by using both lossy and lossless compression techniques. Lossy compression is a method for removing spatial and temporal references. In this paper, we exploit the error propagation property; that is, spatial and temporal reference properties in a video (H.264) decoder.

#### 3.1.1. Spatial Reference Property

In H.264/AVC, each frame consists of a number of slices, and every slice includes individual coding units, called macroblocks, each of which contains one 8 × 8 luminance (Y) array and two corresponding chrominance (Cb and Cr) arrays. A macroblock may be encoded in the intra- or inter-prediction mode. For a video sequence, the slices in the first frame are always encoded as an I-frame (*i.e.*, an IDR-slice always includes an I-frame), and each macroblock is coded in the intra-prediction mode. The slices in the following frames are often encoded as P- or B-frames, with each macroblock being coded in the intra- or inter-prediction mode. The predictions of the pixels in a macroblock are obtained by the linear interpolation of its adjacent pixels.

**Figure 1:**
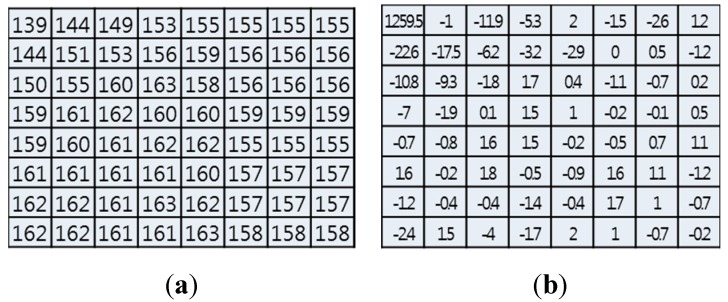

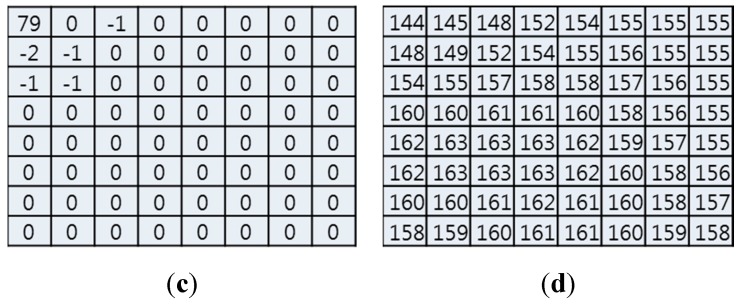
Illustration of intra-prediction mode. (**a**) 8 × 8 macroblock (original image); (**b**) DCT-domain macroblock; (**c**) Quantization-domain macroblock; (**d**) IDCT (reconstructed image).

Figure 1 shows the intra-prediction mode for eliminating spatial redundancy in the lossy compression process of an I-macroblock (*i.e.*, DCT/quantization). An 8 × 8 macroblock of the original image is transformed into the DCT-domain image as shown in Figure 1a,b. Then, the DCT domain is quantized as shown in Figure 1c, and many zeros can be compressed with lossless compression techniques such as Context-Adaptive Variable-Length Coding (CAVLC) or Context-Adaptive Binary Arithmetic Coding (CABAC). In Figure 1d, an 8 × 8 macroblock of the image can be reconstructed by using Inverse DCT (IDCT). As a result, this spatial redundancy can be removed with the intra-prediction mode in order to achieve the aim of compressing the video information.

#### 3.1.2. Temporal Reference Property

**Figure 2:**
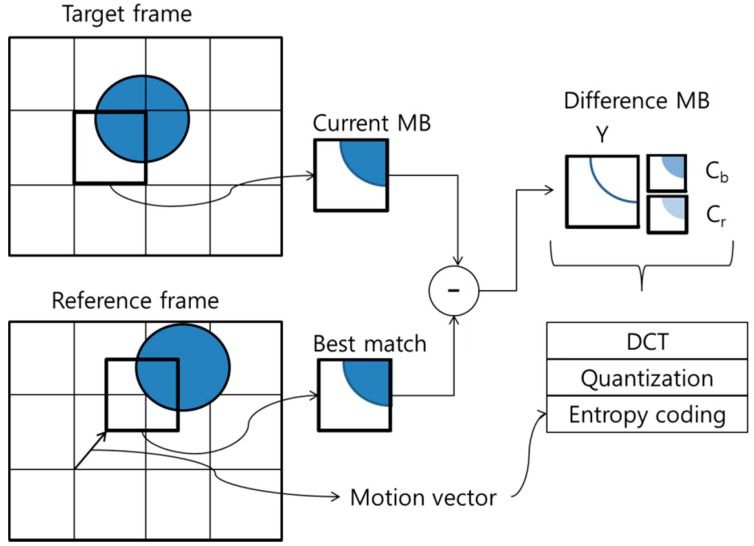
Illustration of inter-prediction mode.

In a P-macroblock, each partitioned block is predicted by a region from the previously coded reference picture; in a B-macroblock, each partitioned block is predicted by either one region or two regions, from one or two previously coded reference pictures, respectively. Figure 2 shows an inter-prediction mode for eliminating temporal redundancy. A P- or B-macroblock (*i.e.*, current MB) finds the best matching macroblock, and the difference MB (*i.e.*, difference between the current MB and the best match MB) and the MV are coded with DCT/quantization/entropy coding. Therefore, the inter-prediction mode can efficiently compress video information by eliminating temporal redundancy.

### 3.2. Selective Encryption of Video for I- and P-/B-Macroblocks

In both intra- and inter-modes, the current MB refers to the previous frames. Thus, if the referenced information (the previous macroblock and pixels) was lossy, the H.264 cannot decode the compressed video data. The aim of the proposed method is to lessen the workload of encrypting the macroblocks of the I- and P-frame, excluding the header, by exploiting the error propagation property of the H.264 standard. Note that, in order to ensure format compliance and security, the frame header is not encrypted. A potential attacker who does not have the required key would need to decode/decompress the transmitted data in order to access the video content. If some macroblocks in each frame are distorted by encrypting it in the encoding/compressing process, the decoder regards the macroblocks as an error, and the error propagates to the successive macroblocks in the frames. To exploit the error propagation property of the H.264 standard, we encrypt only the DC/ACs of I-macroblocks and MVs of P-macroblocks.

#### 3.2.1. I-Macroblock

**Figure 3:**
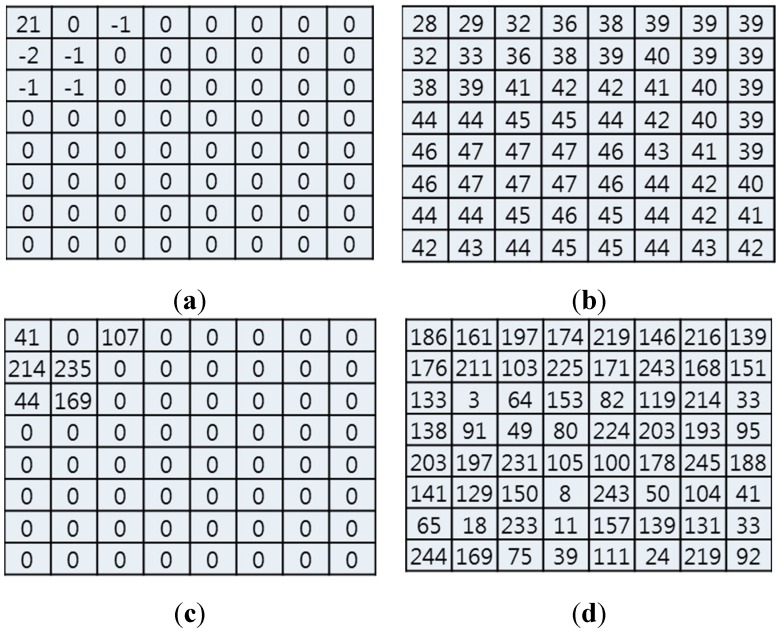
Effect of encrypted DC and DC/AC. (**a**) Encrypted DC; (**b**) Effect of DC (reconstructed image); (**c**) Encrypted DC/AC except zeros; (**d**) Effect of DC/AC (reconstructed image).

In the I-macroblock, encrypted DC or DC/ACs affects the reconstructed image as shown in Figure 3. If the DC is encrypted (*i.e.*, 79 is encrypted to 21), the reconstructed image is distorted by the error propagation as shown in Figure 3a,b. However, the reconstructed pixel pattern still appears in the DC encryption.

In contrast, if DC/ACs except zeros are encrypted (*i.e.*, 79, −1, −2, −1, −1, and −1 are encrypted to 41, 107, 214, 235, 44, and 166, respectively), the reconstructed image is distorted without the pixel pattern as shown in Figure 3c,d. In this case, since we encrypt only six values, the encryption process can provide not only an effective visual security level by error propagation but also fast processing speed. Therefore, we encrypt the DC/ACs of the I-macroblock except zeros.

#### 3.2.2. P- or B-Macroblock

**Figure 4:**
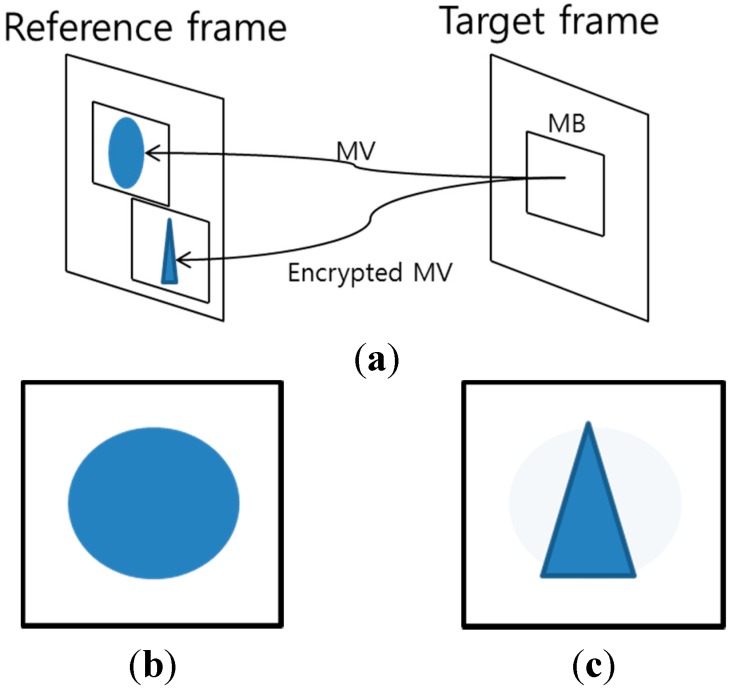
Effect of encrypted motion vector. (**a**) Illustration of encrypted MV; (**b**) Reconstructed image with correct MV; (**c**) Reconstructed image with incorrect MV.

Figure 4 shows the effect of MV encryption in the P- or B-macroblock. The current MB refers to the reference frame with MV and then reconstructs the compressed image (*i.e.*, circle) as shown in Figure 4a,b. However, if the MVs of the current MB are encrypted, the current MB refers to the incorrect region (*i.e.*, triangle), and thus the reconstructed image is distorted as shown in Figure 4a,c. In this case, the image is reconstructed by a composition of the difference MB (*i.e.*, circle) and the incorrect image (*i.e.*, triangle), as shown in Figure 4c. Therefore, we encrypt the MVs of P-or B-macroblocks.

**Figure 5:**
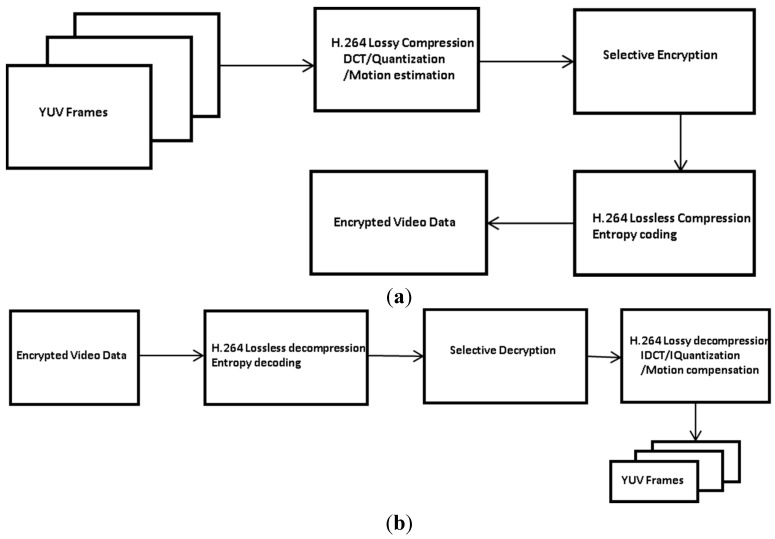
Execution flow of the encryption and decryption processes. (**a**) Compression/encryption process; (**b**) Decompression/decryption process.

Figure 5 shows the execution flow of the encryption and decryption processes. First, frames consisting of YUV are compressed by the H.264 lossy compression (*i.e.*, DCT, quantization, and motion estimation), and the DC/ACs of I-macroblocks except zeros and the MVs of P- or B-macroblocks are encrypted. Then, the H.264 lossless compression (*i.e.*, CAVLC or CABAC) compresses the encrypted video data. Note that, since we encrypt only the information of non-zeros, the compression rate is not degraded by the H.264 encoder.

At the decoder, the encrypted video data are decoded by the H.264 lossless compression. Then, the DC/ACs of I-macroblocks except zeros and the MVs of P- or B-macroblocks are decrypted. Finally, the YUV data are reconstructed by the H.264 lossy decompression. Note that the selectively encrypted video data can be decompressed without a secrete key because the headers are not encrypted, and thus the encrypted video stream does not violate the format compliance requirement. Therefore, the proposed method can ensure the format compliant video compression by using the standard encryption algorithm.

### 3.3. Video Authentication

**Figure 6:**
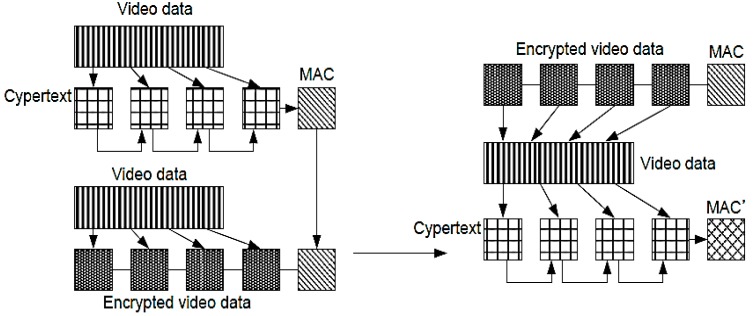
Illustration of the authentication procedures for compressed video data.

In this paper, we apply the AES-CCM in order to ensure both the confidentiality and the integrity of video data. Figure 6 illustrates the authentication procedures for video data, which are compressed and encrypted using H.264 and AES-CTR, respectively, while MAC is generated by the CBC mode.

Encrypting the video data using AES-CTR ensures the confidentiality of the data. To verify MAC, the video data are decrypted using AES, and MAC′ is generated by the CBC mode. Finally, if MAC and MAC′ are exactly identical, we can confirm that the video data are not forged. Note that although MAC is generated at least once for one frame, the data overhead is negligible because of the small size of MAC.

### 3.4. Improving Collective Performance

Since the primary objective of this research is to provide secure transmission of I-frames, the difference between the original image and the decoded image without using the required key should be emphasized. Therefore, the confusion degree (*i.e.*, *CD*) can be defined as the difference between the cipher text and the plaintext in a cryptographic system [29]. In fact, the visual security has been represented as PSNR or MSE in some researches for selective encryption. However, we quantitatively represent the visual security as confusion degree (*CD*), which is similar to [28,29,30,31,32]. In this paper, the structural similarity (*i.e.*, SSIM) [41] is applied in order to quantitatively measure this *CD*. The SSIM metric is calculated with various windows of an image. The measures between two windows *x* and *y* are as follows: μ_*x*_ and μ_*y*_ (the average), $\sigma_{x}^{2}$ and $\sigma_{y}^{2}$ (the variance), *σ_xy_* (the covariance). *c_1_* and *c_2_* (two variables to stabilize the division with a weak denominator). For the *M × N* image size, *CD* can be represented as Equation (1):

(1) $$CD(x,\;y)\;=\;\overset{N}{\underset{y=1}{\sum }}\overset{M}{\underset{x=1}{\sum }}\frac{\left(2\mu_{x}\mu_{y}+c_{1})(2\sigma_{xy}+c_{2}\right)}{\left(\mu_{x}^{2}+\mu_{y}^{2}+c_{1})(\sigma_{x}^{2}+\sigma_{y}^{2}+c_{2}\right)}$$

In order to evaluate the visual security of selective encryption compared with full encryption, visual security (*i.e.*, *VS*) can be defined as in Equation (2). That is, the similarity between the selectively encrypted image and the ideal (full) encrypted image is determined quantitatively:

(2) $$VS\;=\;\frac{Full\;encryption^{\prime }s\;CD}{Selective\;encryption^{\prime }s\;CD}$$

Further, the speedup (*i.e.*, *SP*) of selective encryption compared with full encryption can be defined as Equation (3). Note that *SP* is defined with the encrypted data size, and thus it is a machine-independent definition:

(3) $$SP\;=\;\frac{Full\;encryption^{\prime }s\;data\;size}{Selective\;encryption^{\prime }s\;data\;size}$$

To control *VS* and *SP*, we define the encryption parameter *en*. That is, every *en-*th P-macroblock is encrypted in a total of P-macroblocks. For example, if we set *en* as 2, every second macroblock is encrypted. Note that, *en* = 0 is the full encryption, and *en* = 1 is the selective encryption of all P-macroblocks. Further, *VS* is decreased with increased *en*, but the *SP* is increased.

Finally, to evaluate the collective performance (*i.e.*, *CP*) with *en*, *CP* is represented as a product of *VS* and *SP*, as shown in Equation (4). Note that we can maximize *CP* by analyzing the tradeoff between the visual security level and the processing speed with respect to *en*. Depending on the application, *CP* can be defined by using weight of *VS* or *SP*. We set the weight ratio as 1:1 in this paper:

*CP*(*en*) = *VS*(*en*) *×SP*(*en*) (4)

## 4. Experimental Results

In this paper, a dual-core PC (3.99 GHz, RAM 2.0 GB) was used for the experiments, and the execution times for various handheld devices from the specifications of three devices (*i.e.*, MSP430F1611 [42], ADSP-BF533 [43], and TMS320C6414T [43]) were estimated. The size of the experimental video image was 352 *×* 288, and the frame rate was 30 frames per second (*i.e.*, a total of 300 frames, consisting of two I-frames and 298 P-frames). The total size of the frames was 4.5 MB, including headers, and there were 2 I-frames and 298 P-frames.

Table 1 shows the encrypted data size and *CD* (*i.e.*, SSIM) with *en*. In the video frames (*i.e.*, a total of 300 frames), the number of I- and P-macroblocks were 486 and 17441, respectively. Note that, these macroblocks were selectively encrypted and the size of the encrypted data depended on *en*. In the full encryption (*i.e.*, *en* = 0), since I- and P-macroblocks were compressed using lossless compression, these macroblocks were encrypted in the compressed data. Thus, the size of full encryption was 1000 KB (*i.e.*, uncompressed data size was 4.5 MB). For *CD*, we measured the I- and P-frame. In the experimental results, we found that the effect of the P-macroblock encryption was more than that of the I-macroblock encryption. That is, since the number of I-frames was 2, the *CD* depended on the large number of P-frames (*i.e.*, 298). I- and P-macroblocks were encrypted with the proposed selective encryption where *en* was between 1 and 10. For example, if we set *en* as 5, every fifth P-macroblock was encrypted, and the encrypted data size and *CD* were 30.85 KB and 0.28, respectively.

**Table 1:** Encrypted data size and *CD* with *en*.

Encryption Parameter (*en*)	Encrypted Data Size			*CD* (I-Frames and P-Frames)
	No. of I-Macroblocks	No. of P-Macroblocks	Total Size (KB)	
0	-	-	1000	0.18
1	486	17,441	155.34	0.24
2	486	8742	78.28	0.25
3	486	5819	51.52	0.27
4	486	4367	38.84	0.27
5	486	3369	30.85	0.28
6	486	2902	25.68	0.32
7	486	2463	21.96	0.33
8	486	2166	20.07	0.33
9	486	1987	17.54	0.38
10	486	1801	15.83	0.39

**Figure 7:**
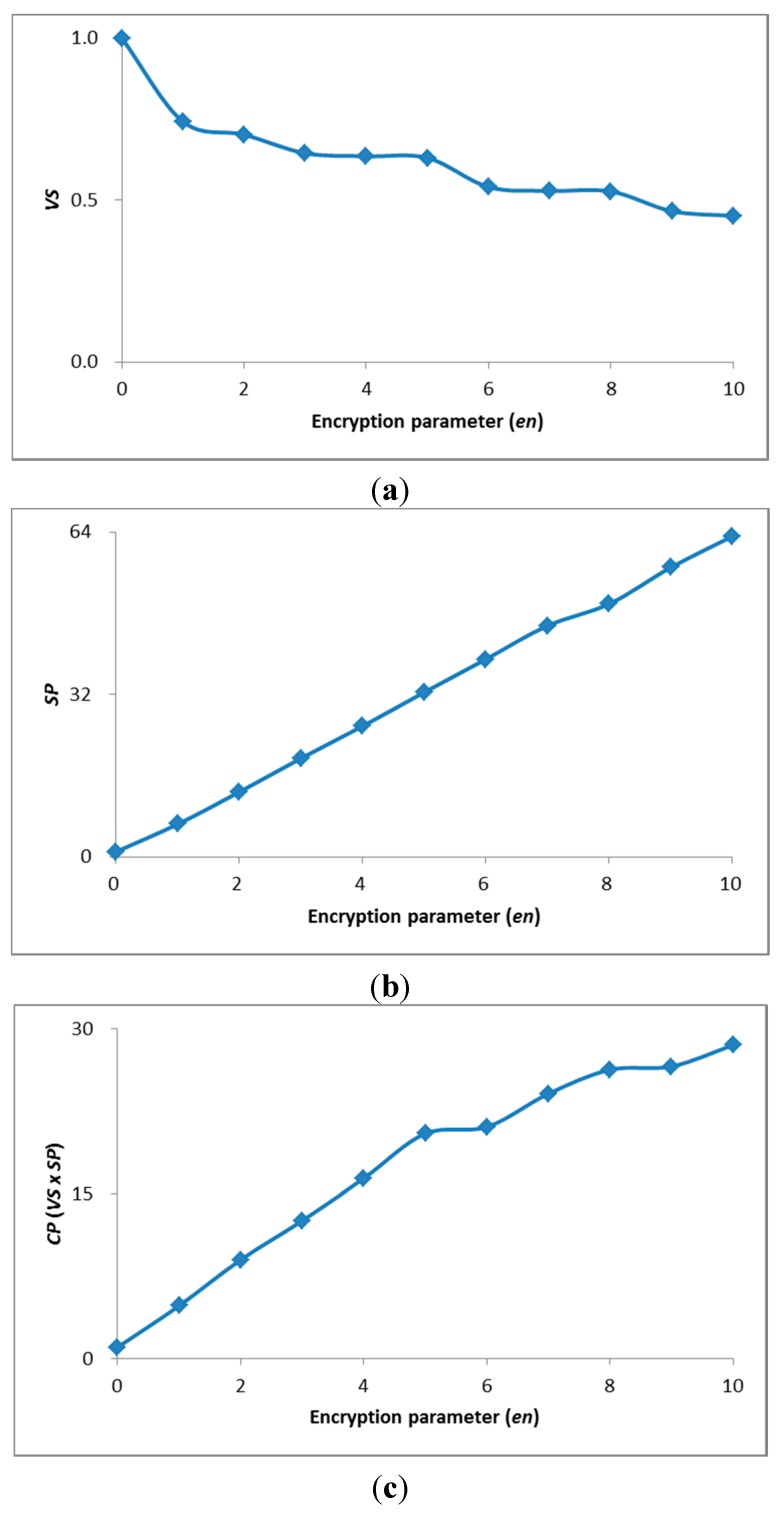
*VS*, *SP*, and *CP* with *en.* (**a**) *VS* with *en*; (**b**) *SP* with *en*; (**c**) *CP* with *en*.

Figure 7 shows *VS*, *SP*, and *CP* with *en*. *VS* was degraded by the increased *en* as shown in Figure 7a, because of an increased *CD*. In contrast, the encrypted data size was decreased by the increased *en*, and thus *SP* improved, as shown in Figure 7b. To provide the high processing speed, *en* should be increased, but the *VS* would be degraded. To avoid considerable degradation of *VS* with increased *en*, we set *en* under the condition of *VS* > 0.5. Figure 7c shows *CP* with *en*. In this case, we can maximize *CP* at *en* = 8, and *SP* was improved by a factor of 49 without any significant degradation of *VS* (*i.e.*, >0.5).

**Figure 8:**
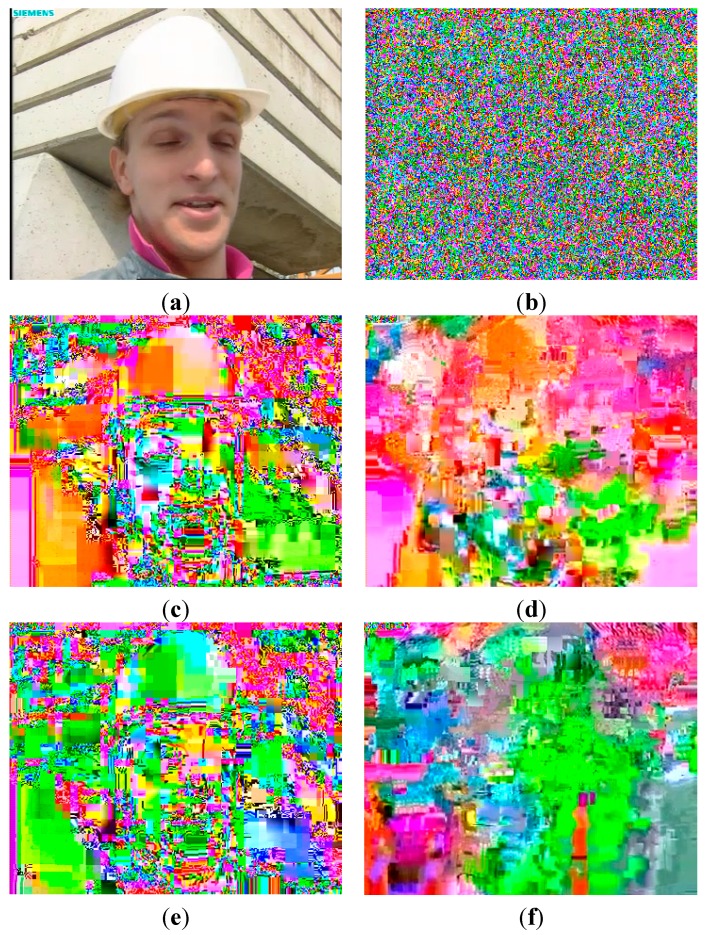
Comparison of full encryption and selective encryption. (**a**) Original; (**b**) Compressed full encryption; (**c**) DC/ACs Encryption of I-frame with *en* = 1; (**d**) MVs encryption of P-frame with *en* = 1; (**e**) DC/ACs Encryption of I-frame with *en* = 8; (**f**) MVs encryption of P-frame with *en* = 8.

Figure 8 shows a comparison of full encryption and selective encryption. The original image and the full encryption image are shown in Figure 8a,b, respectively. Further, we compared the I- and P-frame encryption with *en* as shown in Figure 8c–f. Figure 8c,d shows the I- and P-frame encryption at *en* = 1. Since the DC/ACs were encrypted, the image was not completely decoded owing to the error propagation as shown in Figure 8c. Moreover, MV encryption of the P-frame at *en* = 1 is shown in Figure 8d. Since the MVs were encrypted, the P-macroblocks referred not only to the incorrect region of I-blocks but also to the broken I-blocks (*i.e.*, DC/ACs encryption). Figure c,e shows the effect of I-frame encryption at *en* = 1 and *en* = 8, respectively. In the I-frame, the DC/ACs of I-macroblocks were encrypted regardless of *en*. In contrast, P-frame depended on MV encryption with *en*. Figure 8d,f shows the effect of P-frame encryption at *en* = 1 and *en* = 8, respectively. In the case of *en* = 8, unencrypted MVs can refer to the correct region of I-macroblocks. However, since the unencrypted MVs referred to the broken I-macroblocks, the H.264 decoder could not completely reconstruct the original frame, and thus, provided effective visual security.

We compared the performance of the typical selective encryptions (*i.e.*, I-frame encryption [20], P-frame encryption [21], and combined I-/P-frame encryption [22] of [23]) and the proposed encryption. Table 2 shows the comparison of each performance. The collective performance (*i.e.*, *CP*) was improved by a factor of 1.8, 9.6, and 4.4, respectively, compared with [20,21,22] of typical selective encryptions [23]. Therefore, we confirmed that the proposed selective encryption can improve the processing speed without any significant degradation of the visual security level (*i.e.*, *VS* > 0.5).

**Table 2:** Comparison of performance.

Full Encryption		*VS*	*SP*	*CP*
		1.0	1.0	1.0
Selective encryptions	I-frame encryption [20]	0.3	50.2	15.0
	P-frame encryption [21]	0.4	7.2	2.8
	I- and P-frame encryption [22]	0.6	10.1	6.0
	Proposed (*en* = 8)	0.5	49.8	26.9

Table 3 shows the comparison for execution time of proposed method on various handheld devices where the processor speeds were 8 MHz, 756 MHz, and 400 MHz, respectively. In the full encryption, the execution times of PC (*i.e.*, Intel i5 core processor) and three handheld devices (*i.e.*, MSP430F1611 [42], ADSP-BF533 [43], and TMS320C6414T [43]) were 4.99 ms, 2432.63 ms, 25.74 ms, and 48.65 ms, respectively. In contrast, the execution times of proposed method were 0.01 ms, 48.65 ms, 0.51 ms, and 0.97 ms on various devices, respectively. Therefore, we confirmed that the proposed method can efficiently encrypt the video data on the handheld devices.

**Table 3:** Execution times of proposed method on various handheld devices.

	Intel i5core [3900 MHz]	MSP430F1611 [8 MHz]	ADSP-BF533 [756 MHz]	TMS320C6414T [400 MHz]
Full encryption	4.99 ms	2432.63 ms	25.74 ms	48.65 ms
Proposed (*en* = 8)	0.01 ms	48.65 ms	0.51 ms	0.97 ms

## 5. Conclusions

In this paper, we have proposed a new selective encryption method for mobile devices with limited computational resources. The aim of this method was to protect video data while reducing the computational workload and maintaining the required visual security level. To simultaneously ensure format compliance and security, we encrypted only the DC/ACs of I-macroblocks and the MVs of P-macroblocks. In particular, we exploited the error propagation property in an H.264 decoder and analyzed the tradeoff between the visual security level and the processing speed for improving the collective performance. From the experimental results, the collective performance (*i.e.*, *CP*) was improved, compared to typical selective encryptions. Moreover, we confirmed that the proposed method can significantly reduce the workload of the full encryption method by a factor of 49.8 without any significant degradation of visual security (*i.e.*, *VS* > 0.5).
